# Dibutyl phthalate induces sarcopenia via TNFα/TNFR1-mediated proteolytic and pyroptotic axes: evidence from NHANES and experimental models

**DOI:** 10.3389/fimmu.2026.1853039

**Published:** 2026-06-12

**Authors:** Xiangjiao Yi, Junyan Li, Xintong Long, Xueqin Hu, Lie Yi, Liying Liu, Xiheng Lu, Taotao Xu, Yun Chen, Hou-Feng Zheng, Hongfeng Ruan, Jianguo Tao

**Affiliations:** 1 School of Pharmaceutical Sciences, Zhejiang Chinese Medical University, Hangzhou, Zhejiang, China; 2 Center for Biobased Materials, Muyuan Laboratory, Zhengzhou, Henan, China; 3 The First Affiliated Hospital of Zhejiang Chinese Medical University (Zhejiang Provincial Hospital of Traditional Chinese Medicine), Hangzhou, Zhejiang, China; 4 School of Life Sciences, Westlake University, Hangzhou, Zhejiang, China; 5 Department of Encephalopathy (Department of Neurology), Yunnan Provincial Hospital of Traditional Chinese Medicine, Kunming, Yunnan, China; 6 School of Stomatology, Zhejiang Chinese Medical University, Hangzhou, Zhejiang, China; 7 Suzhou Laboratory of Precision Health and Data Science, The Second Affiliated Hospital of Soochow University, Suzhou, Jiangsu, China; 8 Institute of Health Data Science, Soochow University, Suzhou, Jiangsu, China

**Keywords:** dibutyl phthalate, morroniside, NHANES, pyroptosis, sarcopenia, TNFα, ubiquitin-proteasome

## Abstract

Environmental exposure to plasticizer dibutyl phthalate (DBP) is increasingly implicated in skeletal muscle decline, yet the effects and underlying mechanisms remain elusive. This study investigates the impact of DBP on skeletal muscle using a cross-scale integration of epidemiological modeling, computational toxicology, and experimental validations. Mixture modeling of 3,514 NHANES adults (2011–2018) demonstrated that combined phthalate exposure negatively correlated with skeletal muscle mass not only in aged but also in young populations. DBP metabolite monobutyl phthalate (MBP) emerged as the predominant toxic driver, mediated by inflammation and oxidative stress (Uric acid to High-density lipoprotein cholesterol Ratio, 20.8%). Phenotypically, *in vitro*/*in vivo* models showed that DBP exposure impairs myogenic differentiation, drives transition from oxidative-glycolytic type IIA fibers toward glycolytic type IIB fibers, and depletes regenerative Pax7^+^ satellite cells, accompanied by myofiber atrophy and lipid infiltration, mirroring environmentally-induced myosteatosis. Mechanistically, systems-level analyses and molecular docking suggest a predictive model wherein DBP/MBP could act as pseudo-ligands that dock into the active pocket of the primary trigger TNFα, which specifically upregulates TNFR1 (but not TNFR2), driving dual pathological axes: a proteostatic collapse (ubiquitin-proteasome overactivation and autophagy) and GSDMD-dependent pyroptosis. Pharmacological intervention with Morroniside successfully inhibited TNFα-driven dual axes, restoring homeostasis and alleviating DBP-induced atrophy. Ultimately, our findings expand the traditional paradigm of sarcopenia beyond age-related decline and nutritional deficits, establishing it additionally as an environmentally-driven metabolic pathology and a pressing public health risk. Furthermore, we redefine phthalate toxicity from generalized endocrine disruption to a targeted, receptor-mediated event driven by the TNFα/TNFR1 axis, culminating in environmental sarcopenia.

## 1 Introduction

Sarcopenia, a widespread skeletal muscle disorder characterized by a continuous depletion of muscle mass, diminished strength, and/or compromised physical performance (1), is quickly escalating into a severe challenge for worldwide public health (2). A meta-analysis comprising 49 cohorts indicates that reduced muscle mass correlates with a 36% increase in all-cause mortality (3). Specifically, this condition exacerbates mortality risks associated with cardiovascular diseases and oncology (4). Traditionally viewed as an aging phenotype or a consequence of malnutrition and inactivity, this classical etiology fails to fully explain the rising prevalence of muscle atrophy in non-sedentary and younger populations (5). Emerging evidence suggests that environmental pollutants act as silent, active accelerators of muscular decline (6).

Among tens of thousands of endocrine and metabolic disruptors, phthalate esters (PAEs), particularly dibutyl phthalate (DBP), have become a central focus of intense attention in toxicology and environmental health (7). As an extremely common plasticizer, DBP is widely added to polyvinyl chloride (PVC) plastic products, construction adhesives, printing inks, and an extensive range of cosmetic formulations and personal hygiene items (8). Occupational and environmental monitoring data indicate that human exposure to DBP is nearly ubiquitous and unavoidable (8, 9). For instance, in certain cosmetics like nail polish, DBP concentrations have been detected at alarmingly high levels of up to 24,304 μg/g (10). Such high-concentration topical exposure creates a persistent systemic toxicity risk network in the general population through multiple pathways, including dermal absorption, respiratory inhalation, and dietary ingestion.

The current toxicological framework generally classifies DBP and its primary metabolic metabolite, mono-n-butyl phthalate (MBP), as endocrine disruptors (11). A substantial body of literature has established robust associations between PAEs exposure and widespread metabolic dysfunctions, including type 2 diabetes, insulin resistance, and obesity (12–15). Recent epidemiological studies have linked phthalate exposure to muscle decline, yet they exhibit significant methodological limitations. Previous research relying on older cohorts (e.g., NHANES 1999-2006) (16) emphasized outdated exposure profiles, failing to capture the contemporary chemical burden. Furthermore, many studies relied heavily on binary clinical diagnoses of sarcopenia (17) or functional proxies (e.g., grip strength (18), walking speed (19). These approaches obscure continuous subclinical muscle loss, introduce threshold-based gender and age susceptibility biases, and are highly susceptible to reverse causality. Crucially, these observational studies lack concrete mechanistic validation, leaving the specific causal pathways between DBP exposure and muscle degradation largely speculative and unquantified. Whether and how DBP induces muscle wasting remains unclear.

To bridge the substantial gap between macroscopic epidemiological correlations and microscopic cellular damage, it is imperative to combine population-based data with rigorous wet-lab validations. In this study, we propose a comprehensive cross-scale integration of epidemiological modeling, computational toxicology, and *in vivo*/*in vitro* biological models. Using the contemporary NHANES 2011–2018 datasets, we utilized Weighted Quantile Sum (WQS) alongside Bayesian Kernel Machine Regression (BKMR) mixture models to accurately identify monobutyl phthalate (MBP)-the primary metabolite of DBP-as the core driver of continuous muscle loss (measured via Appendicular Skeletal Muscle Mass Index, ASMI). The corresponding mediation analysis provides direction for potential mechanism analysis. Concurrently, through network pharmacology, transcriptomics, and wet-lab experiments, we seek to reveal the toxic effects of DBP on skeletal muscle and elucidate a novel underlying mechanism: supported by molecular docking, DBP and its metabolite could act as pseudo-ligands that hijack the TNFα-TNFR1 signaling axis via “molecular mimicry”. We demonstrate that this specific receptor-mediated interaction triggers a severe catabolic cascade and Gasdermin D (GSDMD)-dependent pyroptosis. By delineating this novel pathway, this research aims to redefine phthalate toxicity from generalized metabolic disruption to targeted TNFα/TNFR1 dual catabolic and pyroptotic axes, providing critical insights and potential pharmacological targets (e.g., Morroniside) for environmentally-induced musculoskeletal disorders.

## 2 Materials and methods

### 2.1 Study population and epidemiological modeling

NHANES 2011–2018 adults (*n*=3,514, aged ≥20 years) with data on 13 urinary phthalate metabolites and DXA-derived appendicular skeletal muscle mass index (ASMI) and sarcopenia were analyzed. To comprehensively evaluate muscle decline, we utilized two distinct analytical frameworks. Sarcopenia (a binary outcome classified via EWGSOP2 criteria) was specifically employed in single-pollutant logistic regression models to assess the increased clinical risk of diagnosis associated with individual metabolites. Conversely, continuous ASMI was utilized in linear regression, restricted cubic splines (RCS), and mixture models including Weighted Quantile Sum (WQS) regression, Quantile G-Computation (qgcomp), and Bayesian Kernel Machine Regression (BKMR). This continuous framework highlights how combined phthalate exposure drives a cumulative, continuous erosion of muscle mass, effectively capturing subtle, early-stage deterioration before it crosses the clinical diagnostic threshold. To prevent multicollinearity distortion, highly correlated DEHP metabolites were merged into a composite variable (∑DEHP) for the qgcomp analysis. Conversely, they were inputted as individual variables in the BKMR model, leveraging its hierarchical variable selection algorithm to identify specific secondary metabolite effects. Mediation analysis examined inflammation and oxidative stress markers (Uric acid to High-density lipoprotein cholesterol Ratio (UHR), Oxidative Balance Score) as potential biological pathways. Detailed epidemiological and statistical methodologies are provided in the **Supplementary Material**.

### 2.2 Animal experiments

All animal experiments were approved by the Institutional Animal Care and Use Committee of Zhejiang Chinese Medical University (IACUC-20220103-02). Five-week-old male C57BL/6 mice were maintained under specific pathogen-free conditions and randomly assigned to three groups (n = 6 per group): vehicle control (corn oil), DBP-L (50 mg/kg), and DBP-H (250 mg/kg; CAS: 84-74-2). DBP was administered by intragastric gavage 5 days per week for 9 weeks.

To effectively model the lifelong cumulative burden associated with environmental DBP exposure within a compressed short-term murine timeframe, dosages were strategically selected based on established toxicological thresholds. Specifically, the 50 mg/kg/day dose aligns with the recognized No-Observed-Adverse-Effect Level (NOAEL) for subacute tissue toxicity, serving as a rigorous baseline. The 250 mg/kg/day dose was employed as a potent mechanistic probe. Both dosages are consistent with validated experimental protocols previously established (20–22).

At the end of experimental period, all mice were humanely euthanized via gradual-fill CO2 inhalation, with CO_2_ introduced into the chamber at a flow rate of 50% of the chamber volume per minute. Subsequently, skeletal muscle tissues were collected. Tibialis anterior muscles were embedded in OCT compound (Sakura Finetek, Cat#4583), stored at -80 °C, and cryosectioned for histological and immunofluorescence analyses.

### 2.3 Cell culture, differentiation, and reactive oxygen species quantification

We utilized murine C2C12 myoblasts (ATCC, CRL-1772) to assess skeletal muscle toxicity *in vitro* (23). The cells were cultured in medium composed of DMEM with 10% FBS. We initiated myogenesis by DMEM containing 2% horse serum. Following differentiation, the myotubes were subjected to varying concentrations of Dibutyl phthalate (0, 0.1, 0.3, and 1 mM). Based on our previously validated protocols (24), select groups received concurrent interventions of either N-acetyl cysteine (NAC at 2.5 mM) or morroniside (Mor at 80 μg/mL). To maintain stable exposure conditions, the culture media were replenished at 12- to 48-hour intervals. RT-qPCR samples were harvested between 12 and 48 hours to evaluate temporal transcriptional shifts; MyHC immunofluorescence staining was performed at 72–120 h to evaluate myotube formation. Intracellular ROS levels were measured at 24 h using a commercial assay kit (cat# S0033S, Beyotime, China).

### 2.4 Histopathological and immunofluorescence assessments

Tissue cryosections were fixed, then processed for H&E and Oil Red O/hematoxylin staining. We recorded images via a Nikon Ni-E microscope and calculated the TA myofiber cross-sectional area (CSA) utilizing ImageJ (NIH). For immunofluorescence mapping, sections were simultaneously blocked and permeabilized at room temperature (RT) in PBST. Slides were then incubated overnight at 4 °C with primary antibodies against MyHC-IIA (SC-71), MyHC-IIB (BF-F3), Pax7 (Pax7) (all from DSHB), and laminin (Sigma L9393). We applied Alexa Fluor 488- or 568-conjugated secondary antibodies (Invitrogen A21042/A21124) for 1 h at RT. Cultured *in vitro* myotubes were similarly stained using anti-MyHC (R&D Systems MAB4470) followed by an Alexa Fluor 488 secondary (Invitrogen A11001). After mounting with DAPI-inclusive VECTASHIELD (Vector Laboratories H-1200-10), samples were examined under Olympus IX83 or FV3000 microscopes. ImageJ facilitated the quantification of myotube diameter, fusion index (>3 nuclei), satellite cell count (Pax7^+^/fiber), fiber proportions, and CSA.

### 2.5 Western blotting

Protein extraction from gastrocnemius tissues was executed utilizing an Automated Sample Rapid Grinder (Jing Xin, China). Tissues were homogenized. Following a centrifugation to clear cellular debris, we quantified total protein yields utilizing a Thermo Fisher BCA assay kit (Cat# 23225). Equal protein aliquots underwent separation via 4-15% SDS-PAGE before being electrotransferred onto PVDF membranes. Membranes were blocked, followed by an overnight incubation at 4 °C with primary antibodies. Targets sourced from Cell Signaling Technology included: RelA (8242), p50 (13586S), RelB (10544S), TNFα (11948T), p-Smad2/3 (8828), Smad2/3 (8685), Stat3 (12640S), Beclin1 (3495S), GSDMD (39754S), and Cleaved Caspase-1 (89332S). Antibodies procured from Santa Cruz Biotechnology included: p52 (sc-7386), MAFbx (sc-166806), MuRF1 (sc-398608), p53 (sc-393031), NLRP3 (sc-134306), TNFR1 (sc-8436), TNFR2 (sc-8041), p-RelA (sc-136548), and p-IκBα (sc-52943). GAPDH (Goodhere, AB-P-R 001) served as the internal loading control. Finally, protein signals were developed using ECL reagents (Thermo Fisher, Cat# 34577) following incubation with HRP-conjugated secondary antibodies (Cell Signaling 7074/7076).

### 2.6 RT-qPCR assays

To harvest total RNA from the cultured C2C12 myotubes, we applied the TRIzol reagent (Invitrogen, Cat# 15596018CN). Following extraction, cDNA synthesis was carried out via the MonScript RTIII All-in-One Mix (Monad, MR05101). Quantitative PCR amplification was subsequently executed on a Jena Qtower384G thermocycler, utilizing the 2 × Universal SYBR Green Fast qPCR Mix provided by Abclonal (Cat# RK21203). Final transcript abundance was evaluated using the 2^-ΔΔCt^ analytical approach, with *Gapdh* serving as the endogenous reference gene.

### 2.7 Transcriptomic sequencing and bioinformatics

We used 3 biological replicates per group for the RNA-seq analysis. RNA-seq was performed by Novogene (China) on the Illumina NovaSeq 6000 platform using libraries prepared with the NEBNext^®^ Ultra™ RNA Library Prep Kit (NEB, USA) after quality assessment on an Agilent Fragment Analyzer 5400. Raw reads were processed on the Galaxy platform, and low-abundance genes (<10 counts in <3 samples) were removed. Differential expression analysis was performed with DESeq2 (Padj < 0.05). GO/KEGG enrichment and GSEA were conducted using Metascape (25) and the GseaVis R package, respectively.

### 2.8 Network pharmacology and molecular docking

Sarcopenia-related targets were retrieved from DrugBank, OMIM, and GeneCards, while candidate DBP targets were predicted using SwissTargetPrediction (Daina et al., 2019). Overlapping targets were identified using jvenn (Bardou et al., 2014). A compound-target-disease network was constructed in Cytoscape (v3.10.3), and hub genes were ranked using the MNC algorithm in cytoHubba. GO and KEGG enrichment analyses were performed using Metascape (Zhou et al., 2019) and visualized with SRplot (Tang et al., 2023). For molecular docking, protein structures were downloaded from RCSB PDB, preprocessed in PyMOL (v3.1.6.1) and AutoDock Tools (v1.5.7), and binding pockets were predicted with P2Rank (Eberhardt et al., 2021; Trott and Olson, 2010). Docking was performed using AutoDock Vina (v1.2.7), and the lowest-energy conformation was analyzed with PLIP (Schake et al., 2025) and visualized in PyMOL.

### 2.9 Experimental statistical analysis

Data are presented in mean ± SD. To evaluate variances across the experimental groups, we applied a one-way ANOVA, subsequently utilizing Tukey’s test to resolve any multiple comparisons. The threshold for statistical significance was established at a *p*-value of less than 0.05 (*p < 0.05, **p < 0.01, ***p < 0.001, ****p < 0.0001). Both the mathematical computing and the creation of visual plots were executed within GraphPad Prism 10.

## 3 Results

### 3.1 Baseline characteristics and independent associations of phthalate metabolites with sarcopenia

The demographic and clinical profiles of the 3,514 subjects analyzed in our cohort (**Supplementary Figure 1**) are detailed in **SupplementaryTable 1**, comprising 3,180 non-sarcopenic individuals and 334 patients with sarcopenia. To identify the specific toxicological contributors to muscle loss, we evaluated the independent links between individual urinary phthalate metabolites and sarcopenia risk. After fully adjusting for demographics, lifestyle, and comorbidities (Model III), multivariable logistic regression revealed that mono-n-butyl phthalate (MBP), the DBP primary metabolite, was the most potent predictor of sarcopenia (OR = 1.37, *P* = 0.016) (**Supplementary Table 2**). Consistent with this, we observed a significant negative linear correlation between MBPand the Appendicular Skeletal Muscle Mass Index (ASMI) (**Supplementary Table 3**; **Supplementary Figure 2**). Subgroup analyses further indicated that the deleterious effect of MBP on sarcopenia wasparticularly pronounced in vulnerable populations, notably individuals aged over 40 years (OR = 1.64, *P* < 0.05) (**Supplementary Table 4**). These robust associations persisted even in sensitivity analyses (**Supplementary Table 5**), firmly establishing MBP as a prominent risk factor for skeletal muscle deterioration.

### 3.2 Mixture effects of phthalate exposure and underlying mediating mechanisms

**Figure 1:**
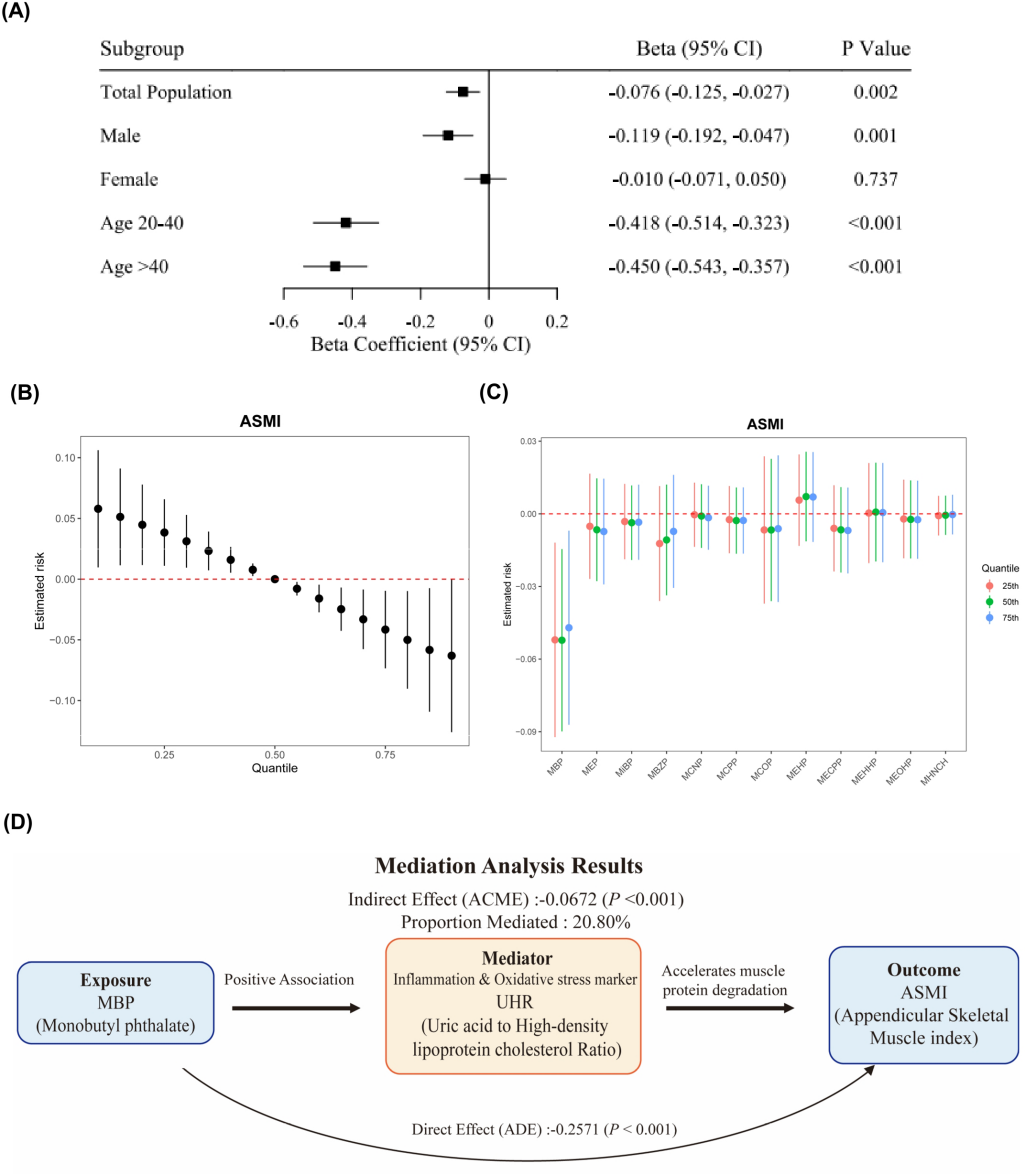
Population-based associations between phthalate exposure and Appendicular Skeletal Muscle Mass Index (ASMI): multi-model assessments and UHR-mediated pathological pathways. **(A)** Forest plot of qgcomp models showing associations of phthalate mixture exposure with ASMI in total and stratified populations, presented as Beta with 95% CIs and *P*-values. **(B)** Overall cumulative effect of the phthalate mixture on ASMI estimated by BKMR; shaded area indicates 95% CI. **(C)** BKMR univariate exposure–response relationships for individual metabolites, shown as estimated risk with 95% CrIs at the 25th, 50th, and 75th exposure quantiles relative to the median. **(D)** Mediation analysis of inflammation and oxidative stress, illustrating a significant pathway in which MBP increases UHR, which in turn promotes muscle atrophy; ACME, ADE, *P*-values, and proportion mediated are shown. All models were adjusted for age, race/ethnicity, BMI, smoking status, and energy intake. CI, confidence interval.

Spearman correlation analysis of the 13 urinary phthalate metabolites revealed that extensivepositive correlations across the biomarkers (**Supplementary Figure 3**). Given that human exposure to phthalates occurs as a complex mixture, we assessed the combined toxicological burden utilizing both Bayesian Kernel Machine Regression (BKMR) and Quantile G-Computation (Qgcomp) methodologies. The Qgcomp model revealed a negative association between the overall phthalate mixture and ASMI (Total β = -0.076, *P* = 0.002) (**Figure 1A**). Crucially, MBP was identified as the predominant negative contributor to this mixture effect, carrying the highest negative weight in the total, male, and young (Age 20-40) population, which was even more exacerbated in the older (Age > 40) demographic (**Figure 1A**; **Supplementary Table 6**). In parallel, the BKMR framework verified a cumulative deterioration of ASMI stemming from the combined chemical exposure (**Figure 1B**; **Supplementary Figure 4A**), and confirmed the high variable importance of specific metabolites, with MBP yielding aremarkably high posterior inclusion probability (**Supplementary Table 7**). Furthermore, the univariate exposure-response plot from the BKMR model illustrated that ASMI decrease with MBP exposure, a trend not observed for the remaining phthalate derivatives (**Figure 1C**). The bivariate exposure-response matrix further demonstrated that the exposure-responserelationship for each phthalate derivative was relatively independent of the concentrations of othermixture components (**Supplementary Figure 4B**). Consistently, MBP drives the overall toxicity of the mixture through its strong independent negative effect. To bridge these epidemiological observations with mechanistic pathways-specifically investigating whether phthalate metabolites induce muscle atrophy via systemic disruption, we conducted mediation analyses. We discovered that the detrimental effects of MBP on ASMI were mediated (20.8%) by the inflammation and oxidative marker UHR (Uric acid to High-density lipoprotein cholesterol Ratio) (26–28) (**Figure 1D**; **Supplementary Table 8**). Together, our findings demonstrate that sarcopenia is not solely a traditional geriatric or nutritional outcome, but can also manifest as an environmentally-related condition, highlighting it as a broad public health risk. Key phthalate metabolite and the broader phthalate mixture may compromise skeletal muscle integrity by shifting the body toward an inflammatory, pro-oxidative state.

### 3.3 DBP exposure drives skeletal muscle atrophy and ectopic lipid deposition

**Figure 2:**
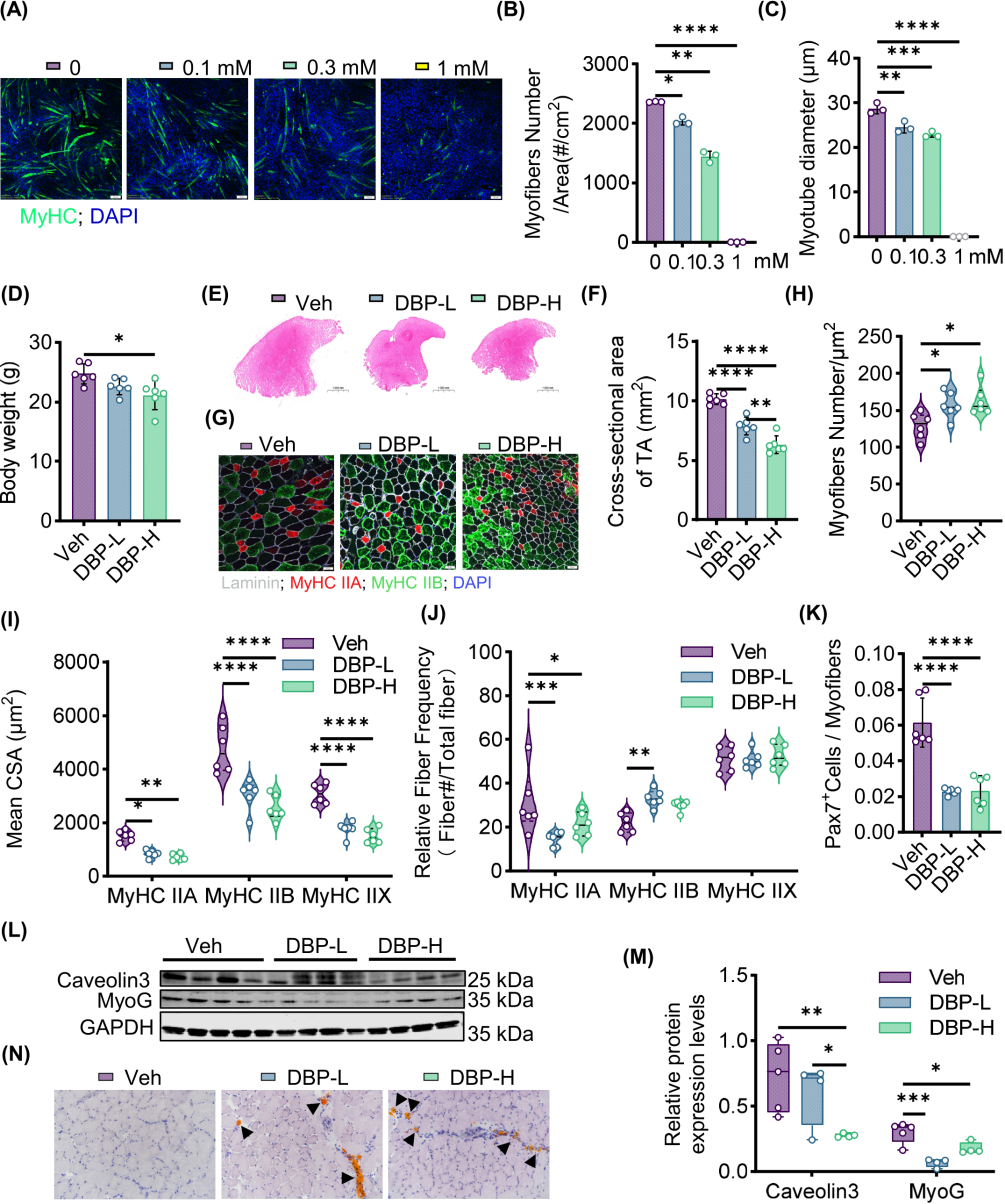
DBP exposure induces skeletal muscle atrophy, suppresses myogenic differentiation, and promotes lipid accumulation. **(A–C)** Effects of DBP on C2C12 myotube formation at 72–120 hours. **(A)** Representative MyHC (green) and DAPI (blue) images. Scale bar, 100 μm. Quantification of **(B)** myofiber number per area and **(C)** myotube diameter (*n*=3). **(D–F)** *In vivo* muscle assessment. **(D)** Body weight, **(E)** representative H&E staining of TA sections, and **(F)** TA cross-sectional area (CSA) in Veh, DBP-L (50 mg/kg), and DBP-H (250 mg/kg) groups (*n*=6/group). **(G–J)** Fiber-type analysis. **(G)** Representative staining of laminin (white), MyHC IIA (red), MyHC IIB (green), and DAPI (blue) in TA sections. Scale bar, 50 μm. Quantification of **(H)** myofiber density, **(I)** fiber-type-specific CSA, and **(J)** fiber frequency. **(K)** Pax7^+^ cells per myofiber. **(L, M)** Western blot analysis of Caveolin3 and MyoG, including **(L)** representative bands and **(M)** quantification relative to GAPDH. **(N)** Representative Oil Red O staining showing intramyocellular lipid accumulation (arrowheads). Data are mean ± SD. One-way ANOVA with Tukey’s *post hoc* test. * *p* < 0.05, ** *p* < 0.01, *** *p* < 0.001, and **** *p* < 0.0001 vs. 0 mM or Vehicle group.

To corroborate our epidemiological findings, we characterized the toxic phenotype using both *in vitro* and *in vivo* models. In C2C12 myoblasts, DBP treatment impaired myogenic differentiation. Immunofluorescence staining revealed a marked reduction in myotube marker Myosin Heavy Chain (MyHC) positive areas (**Figure 2A**). Quantitative analysis confirmed a dose-dependent decline in the myofiber number (**Figure 2B**) and myotube diameters (**Figure 2C**). Consistent with our *in vitro* observations, mice exposed to high-dose DBP exhibited lowered body weight (**Figure 2D**). Histological assessment of the Tibialis Anterior (TA) muscle highlighted structural deterioration. Specifically, the cross-sectional area (CSA) of muscle fibers diminished notably (**Figure 2E**), as confirmed by statistical quantification (**Figure 2F**). We further quantified muscle fiber composition using immunofluorescence (**Figure 2G**). Although myofiber density appeared elevated (**Figure 2H**), this reflected the denser packing of atrophied fibers rather than growth. Indeed, the mean CSA across MyHC IIA, IIB, and IIX fiber types decreased universally (**Figure 2I**), echoing the results in **Figures 2E, F**. As illustrated in **Figures 2G, J**, the proportion of type IIX fibers remained unaffected by DBP treatment. However, the exposure led to a decline in mixed oxidative/glycolytic type IIA fibers, alongside a concurrent rise in fast-glycolytic type IIB fibers. Consequently, these findings indicate that DBP promotes a transition toward a predominantly glycolytic muscle fiber profile, a change that could potentially weaken fatigue endurance. Additionally, the pool of Pax7+ satellite muscle stem cells decreased following exposure (**Figure 2K**), indicating compromised regenerative potential. Molecular analysis validated these histological observations. Western blotting demonstrated the downregulation of critical skeletal muscle architecture and differentiation markers, Caveolin3 and MyoG (29, 30) (**Figures 2L, M**). Oil Red O staining, a method for visualizing lipid accumulation (31), detected extensive abnormal lipid infiltration within inter-muscular spaces (**Figure 2N**). These data collectively indicate that DBP induces a pathology consistent with sarcopenia.

### 3.4 Molecular mechanisms linking DBP exposure to sarcopenia via network pharmacology and transcriptomics

**Figure 3:**
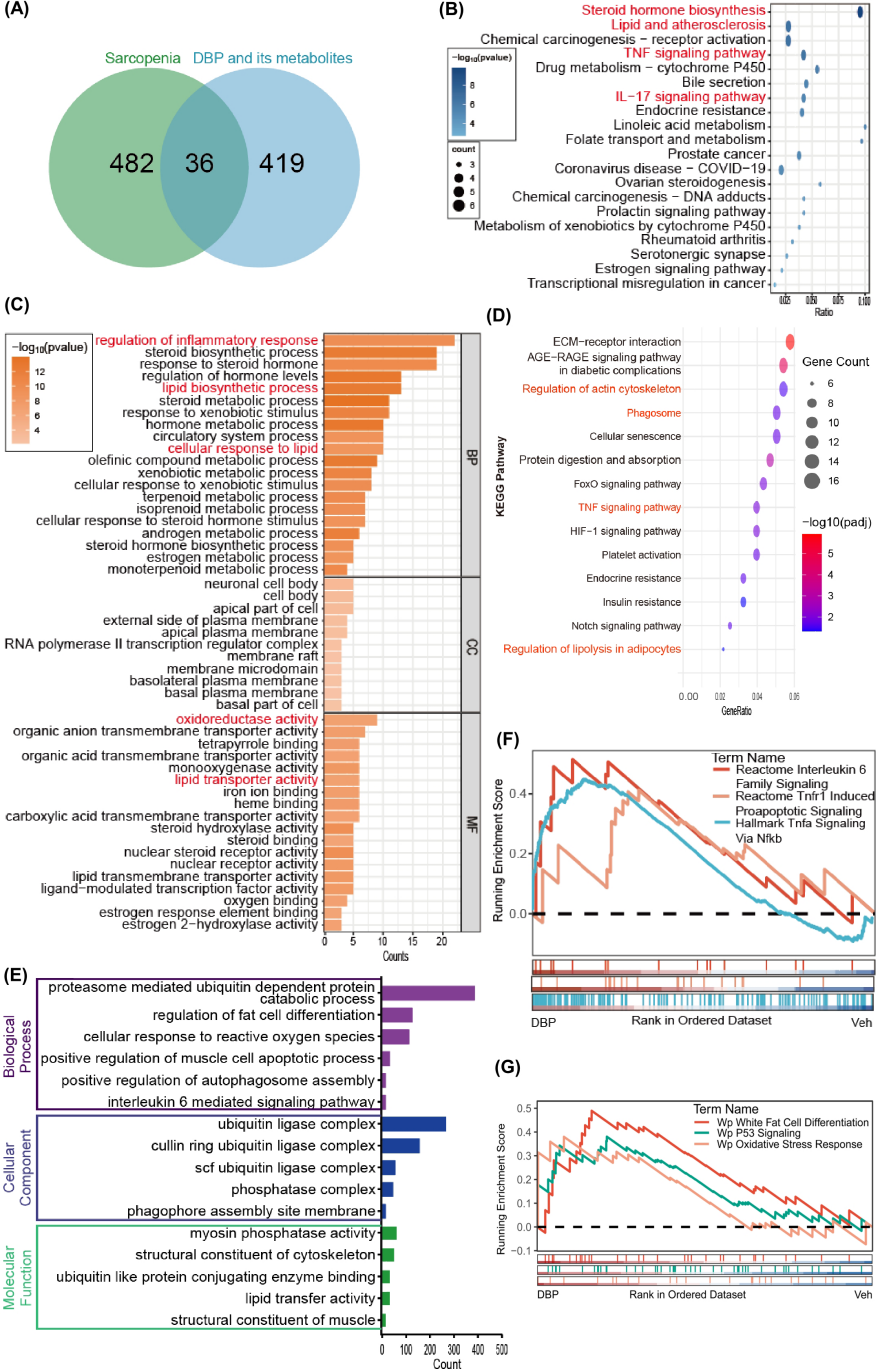
Identification of potential molecular mechanisms linking DBP exposure to sarcopenia via network pharmacology and transcriptomic analyses. **(A–C)** Network pharmacology analysis. **(A)** Venn diagram of sarcopenia-, DBP-, and metabolite-related targets, identifying 36 shared targets. **(B)** KEGG and **(C)** GO enrichment of the overlapping targets (BP, CC, and MF). **(D–G)** *In vivo* transcriptomic validation. **(D)** KEGG and **(E)** GO enrichment of DEGs in skeletal muscle from DBP-treated mice vs. controls. **(F, G)** GSEA plots showing enriched pathways, including **(F)** inflammatory pathways and **(G)** metabolic and structural pathways. n = 3 biological replicates.

We integrated network pharmacology with *in vivo* transcriptomic data to map how DBP drives muscle loss. The intersection of DBP-related toxicity targets and sarcopenia genes identified 36 common targets (**Figure 3A**). Enrichment analyses using KEGG and GO identified the TNF signaling pathway and lipid biosynthetic process as central regulatory hubs (**Figures 3B, C**). Data from the skeletal muscle transcriptome of DBP-exposed mice validated these initial predictions. Differentially expressed genes showed significant enrichment in the TNF signaling pathway (**Figure 3D**). Furthermore, GO analysis revealed a robust activation of the “proteasome mediated ubiquitin dependent protein catabolic process”, pointing to accelerated protein degradation (**Figure 3E**). To understand the specific pathological progression, we used Gene Set Enrichment Analysis (GSEA). On the signaling level, we confirmed a significant activation of inflammatory cascades. Specifically, gene sets for “Tnfa Signaling Via Nfkb” and “Tnfr1 Induced Proapoptotic Signaling” showed strong enrichment in the DBP group (**Figure 3F**). This inflammatory state was coupled with metabolic and structural shifts. GSEA further highlighted the activation of “White Fat Cell Differentiation” and the “Oxidative Stress Response” (**Figure 3G**). These findings suggest that DBP drives sarcopenia by simultaneously triggering inflammation, oxidative stress, protein degradation, and the transition toward a “fat-like” metabolic phenotype.

### 3.5 DBP exposure triggers ubiquitin-proteasome, autophagy, and pyroptosis pathways in skeletal muscle

**Figure 4:**
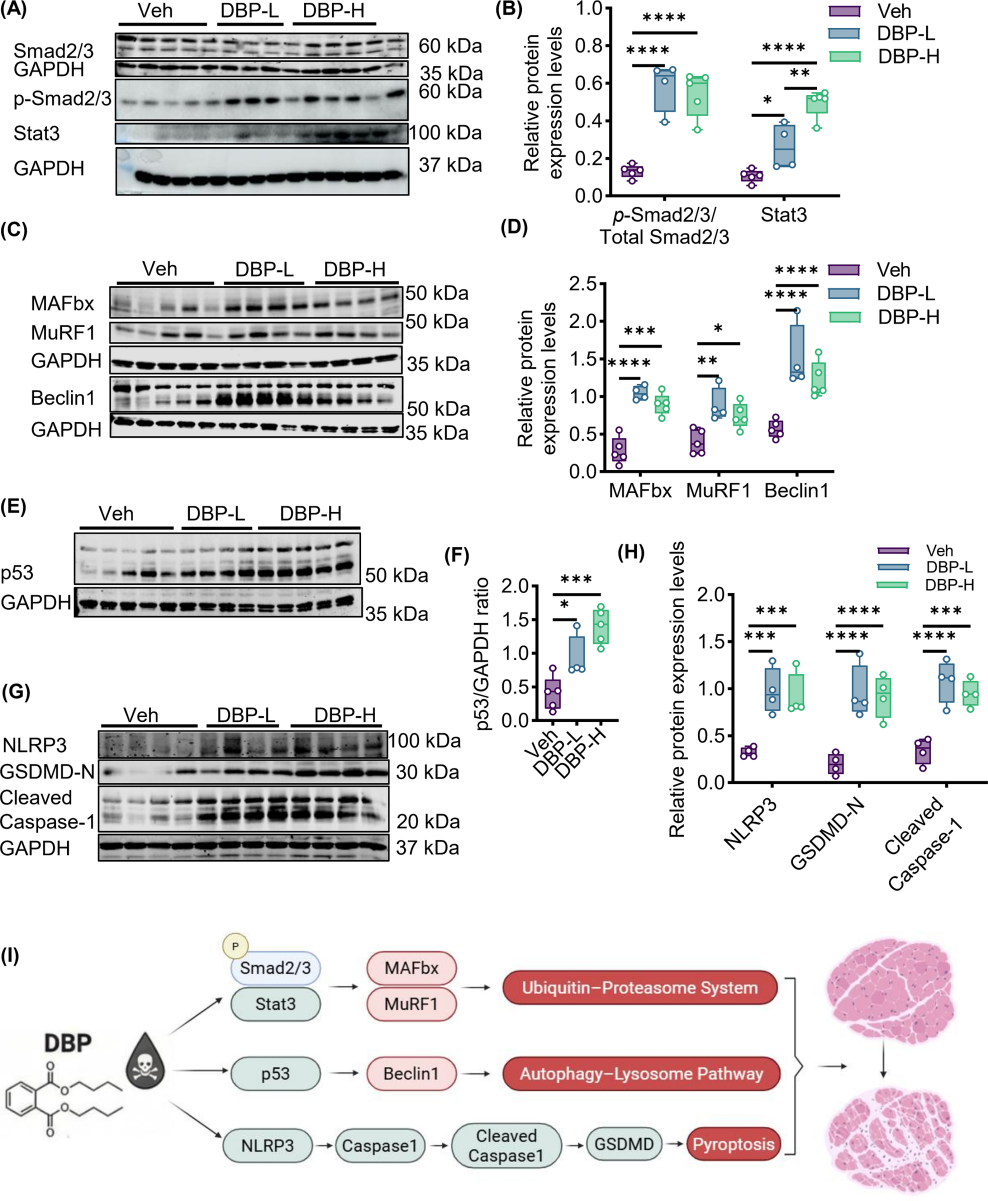
DBP exposure activates ubiquitin-proteasome, autophagy, and pyroptosis pathways in mouse skeletal muscle. **(A–H)** Western blot analysis of key signaling proteins in the gastrocnemius muscle of mice from Vehicle (Veh), Low-dose (DBP-L), and High-dose (DBP-H) groups. **(A, B)** Activation of upstream catabolic signaling. **(A)** Representative blots and **(B)** quantification of p-Smad2/3 relative to total Smad2/3, and Stat3. **(C, D)** Upregulation of muscle atrophy-related E3 ligases and autophagy markers. **(C)** Representative blots and **(D)** quantification of MAFbx, MuRF1, and Beclin1. (Note: As the experiments in **Figure 2L** and **Figure 4C** were conducted using the exact same set of samples, the upper GAPDH loading control image (corresponding to MAFbx and MuRF1) in **Figure 4C** is re-used from **Figure 2L** for illustrative purposes.) **(E, F)** Evaluation of cellular stress response. **(E)** Representative blots and **(F)** quantification of p53 expression. **(G, H)** Activation of NLRP3 inflammasome-mediated pyroptosis. **(G)** Representative blots and **(H)** quantification of NLRP3, GSDMD-N, and Cleaved Caspase-1. **(I)** Proposed mechanism of DBP-induced sarcopenia via Smad2/3- and Stat3-mediated UPS, p53-Beclin1-dependent autophagy-lysosome pathway, and NLRP3-Caspase-1-GSDMD pyroptosis. Created in BioRender. Yi, X. (2026) https://BioRender.com/ov3c5i6. Data are presented as mean ± SD (*n* = 4–5 biological replicates). One-way ANOVA with Tukey’s *post hoc* test. * *p* < 0.05, ** *p* < 0.01, *** *p* < 0.001, and **** *p* < 0.0001 vs. Vehicle group.

We investigated the catabolic signaling axes in the gastrocnemius muscle to identify how DBP executes muscle wasting. Western blot results showed that in mice exposed to DBP, p-Smad2/3 and total Stat3 protein expression increased (**Figures 4A, B**). These changes provide the transcriptional basis for muscle breakdown. The ubiquitin-proteasome system (UPS) appears to be a major downstream target of these regulators. Our data revealed that DBP caused a dose-dependent increase in muscle-specific E3 ligases, specifically MAFbx and MuRF1 (**Figures 4C, D**). We also found a simultaneous induction of p53 and the autophagy marker Beclin1, suggesting that autophagy also contributes to the loss of muscle mass (**Figures 4C–F**). Beyond standard degradation, we identified pyroptosis as a distinct pathogenic mechanism in DBP-induced tissue damage. DBP exposure primed the NLRP3 inflammasome, which triggered the cleavage of Caspase-1. This process resulted in the accumulation of Gasdermin D N-terminal (GSDMD-N) (32), a key executioner of cell pyroptosis (**Figures 4G, H**). The activation of this pathway leads to the release of pro-inflammatory cytokines, further aggravating the local microenvironment (33). Together, these results reveal that DBP triggers sarcopenia through a combined activation of the UPS, autophagy, and pyroptosis axes (**Figure 4I**).

### 3.6 DBP activates the dual TNFα/NF-κB signaling axis: from computational prediction to *in vivo* evidence

**Figure 5:**
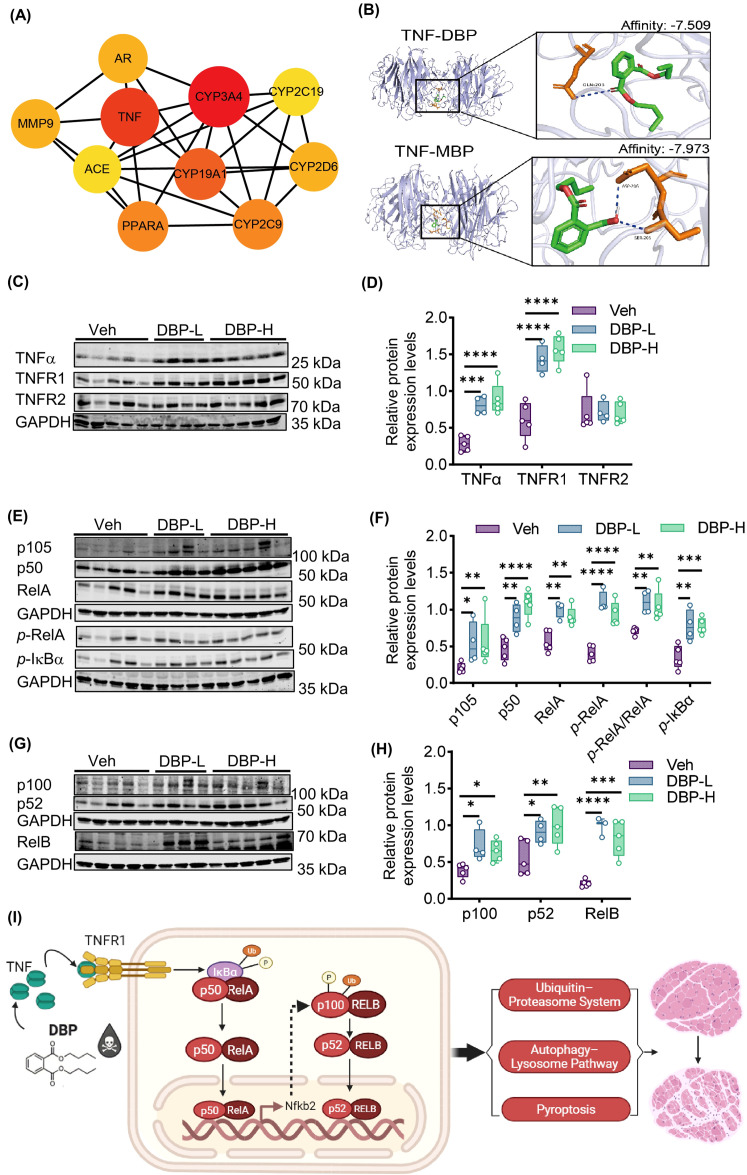
DBP activates TNF-α/NF-κB signaling pathways identified by computational predictionand validated *in vivo*. **(A)** PPI network of 36 shared sarcopenia- and DBP-related targets; the top 10 hub genes (yellow/orange) were identified by the cytoHubba MNC algorithm. **(B)** Molecular docking of TNF with DBP and MBP, showing 3D binding modes, key interactions, and binding affinities (kcal/mol), analyzed by AutoDock Vina and PLIP. **(C–H)** Validation of TNFα/NF-κB signaling in gastrocnemius muscle. **(C, D)** Representative blots and quantification of TNF-α, TNFR1, and TNFR2. **(E, F)** Representative blots and quantification of p105, p50, RelA, p-RelA, and p-IκBα. **(G, H)** Representative blots and quantification of p100, p52, and RelB. **(I)** Proposed mechanism in which DBP activates TNFα/TNFR1 signaling and both canonical (p50/RelA) and non-canonical (p52/RelB) NF-κB pathways, promoting muscle wasting through autophagy, UPS, and pyroptosis. Created in BioRender. Yi, X. (2026) https://BioRender.com/ov3c5i6. Data are presented as mean ± SD (n = 3 biological replicates). One-way ANOVA with Tukey’s *post hoc* test. * *p* < 0.05, ** *p* < 0.01, *** *p* < 0.001, and **** *p* < 0.0001 vs. Vehicle group.

We used an *in silico* strategy to identify the primary molecular targets triggering DBP-induced muscle damage. By constructing a protein-protein interaction (PPI) map from the 36 overlapping targets, we utilized the MNC ranking algorithm to isolate core regulators, highlighting TNF as the primary network hub (**Figures 3A**, **5A**). This network perfectly aligned with our *in vivo* transcriptomic profiling, which showed marked enrichment in the TNF signaling pathway (**Figures 3D, F**), collectively establishing TNFα as the primary trigger in this toxicity network. Subsequent molecular docking simulations revealed that DBP and its metabolite MBP could form stable complexes within the TNF protein’s active pocket via hydrogen bonding (**Figure 5B**). These strong binding affinities suggest that DBP potentially triggers TNF signaling through physical interaction. Our *in vivo* results showed a significant increase in TNFα protein abundance within the gastrocnemius muscles following DBP exposure (**Figures 5C,D**). At the receptor level, we observed a specific activation pattern: TNFR1 levels were markedly elevated, whereas TNFR2 remained unchanged (**Figures 5C, D**). This TNFR1-specific rise triggered a comprehensive response in the NF-κB system. In the canonical pathway, increased p-IκBα released its hold on NF-κB, accompanied by the processing of p105 into p50 and enhanced phosphorylation of RelA (*p*-RelA/RelA) (**Figures 5E, F**). Simultaneously, the non-canonical pathway was activated (34, 35), evidenced by the increased cleavage of p100 into p52 and the significant accumulation of RelB (**Figures 5G, H**). Together, these data suggest that DBP targets TNFα to specifically activate TNFR1, coordinating dual NF-κB pathways to drive muscle decline (**Figure 5I**).

### 3.7 Morroniside confers superior protection against DBP-induced atrophy by dual-targeting oxidative stress and the TNFα-driven inflammatory cascade

**Figure 6:**
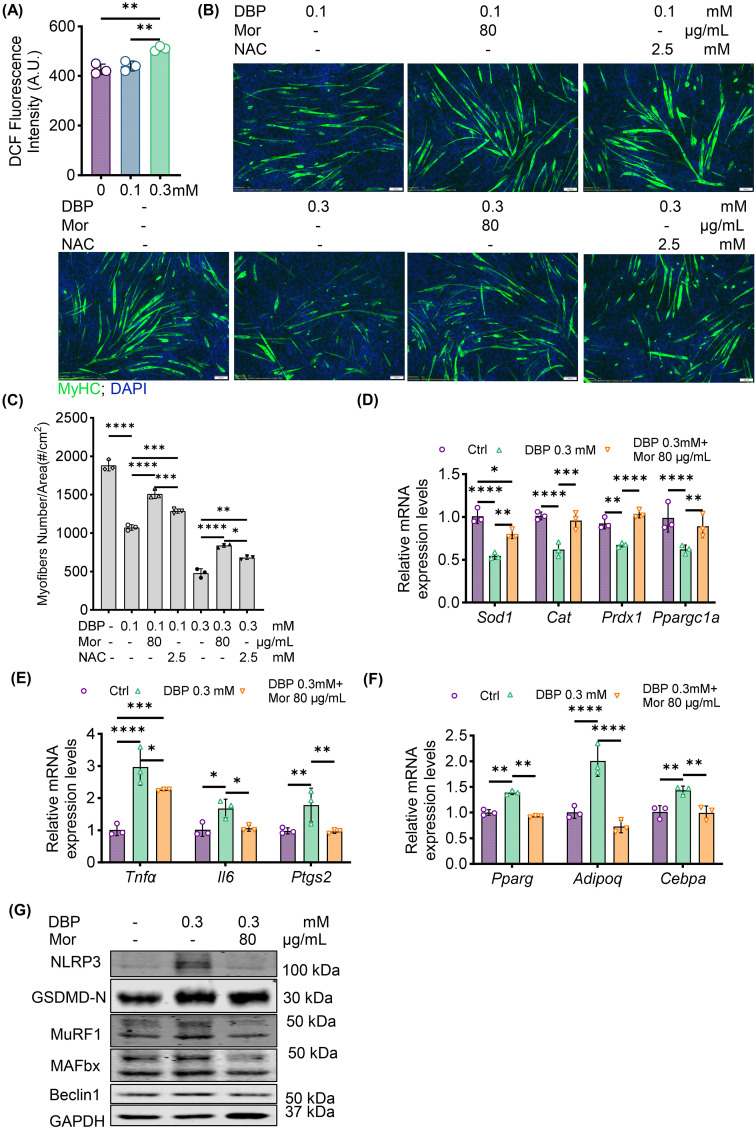
Morroniside confers superior protection against DBP-induced atrophy by dual-targeting oxidative stress and the TNFα-driven inflammatory cascade. **(A)** Intracellular ROS in C2C12 cells treated with DBP (0, 0.1, 0.3 mM) for 24 h, quantified by DCF fluorescence (A.U.); 0.3 mM DBP increased ROS vs. control. **(B, C)** Effects of Morroniside (Mor) and N-acetylcysteine (NAC) on myotube formation at 72–120 hours. **(B)** Representative MyHC (green) and DAPI (blue) immunofluorescence images of C2C12 myotubes treated with DBP (0.1, 0.3 mM) alone or with Mor (80 μg/mL) or NAC (2.5 mM). Scale bar, 100 μm. **(C)** Quantification of myofiber number per area. **(D–F)** qPCR analysis of genes related to **(D)** antioxidant defense and mitochondrial biogenesis, **(E)** inflammation, and **(F)** adipogenesis at 12–48 hours. Mor restored antioxidant gene expression and reduced inflammatory and adipogenic markers. **(G)** Western blot analysis of pyroptosis (NLRP3, GSDMD), atrophy (MuRF1, MAFbx), and autophagy (Beclin1) markers; Mor attenuated DBP-induced increases. Data are presented as mean ± SD (*n* = 3 biological replicates). One-way ANOVA with Tukey’s *post hoc* test. * *p* < 0.05, ** *p* < 0.01, *** *p* < 0.001, and **** *p* < 0.0001.

Given that TNF signaling is a potent inducer of reactive oxygen species (ROS), which in turn can amplify NF-κB signaling, we hypothesized that DBP establishes a self-perpetuating “ROS-inflammation vicious cycle” to drive muscle wasting. To validate this interplay, we first assessed the oxidative status in C2C12 myotubes. DBP treatment triggered an increase in intracellular ROS (**Figure 6A**). We then introduced N-acetylcysteine (NAC, a specific ROS scavenger) and Morroniside (Mor, a compound with dual antioxidant and anti-inflammatory properties (6, 24) to determine if breaking this loop could rescue the phenotype. Immunofluorescence imaging demonstrated that Mor and NAC treatments effectively reversed myotube shrinkage, restoring myotube density and MyHC-positive areas. Notably, Morroniside demonstrated superior protective efficacy compared to NAC (**Figures 6B, C**). Furthermore, transcriptional profiling confirmed that Morroniside broke the feed-forward loop. It not only restored the expression of antioxidant defense genes (*Sod1, Cat, Prdx1*) *(*36) and the mitochondrial regulator *Ppargc1a (*37) (**Figure 6D**), but concurrently suppressed the inflammatory genes driven by the NF-κB pathway (*Tnfa, Il6, Ptgs2*) *(*38, 39) (**Figure 6E**), and preventing the shift toward adipogenesis (*Pparg, Adipoq, Cebpa*) (40) (**Figure 6F**). Finally, Western blot analysis showed that treatment with Morroniside significantly attenuated the overactivation of the pyroptosis axis (NLRP3, GSDMD-N), the ubiquitin-proteasome system (MuRF1, MAFbx), and the autophagy (Beclin1) (**Figure 6G**). Collectively, this suggests that targeting ROS alone (via NAC) provides only partial mitigation, whereas the dual-targeting action of Morroniside offers comprehensive protection. Morroniside inhibits protein degradation and pyroptosis, preventing the sarcopenia phenotype.

## 4 Discussion

Sarcopenia has traditionally been categorized as an geriatric outcome driven by neuromuscular decline and nutritional deficits (41). Epidemiologically, however, we discovered that combined phthalate exposure is associated with decreased skeletal muscle mass not solely in aged but also in young populations. Monobutyl phthalate (MBP), the dibutyl phthalate (DBP) metabolite, was identified as the prominent risk factor with inflammation and oxidative stress mediating this damage. At the cell and tissue level, DBP inhibits myogenic differentiation, exhausts Pax7^+^ satellite cells and drives a shift from versatile oxidative/glycolytic type IIA fibers toward fatigue-susceptible fast-glycolytic type IIB fibers, coupled with lipid infiltration. Mechanistically, we proposed a potential molecular initiating event: DBP and MBP could predictively act as structural pseudo-ligands that dock into the active pocket of primary trigger TNFα protein, specifically hijacking the TNFR1 signaling axis. This molecular mimicry could trigger NF-κB activation, ROS generation, protein degradation and pyroptosis. These findings shift the paradigm of phthalate toxicity from generalized endocrine disruption to targeted, receptor-mediated TNFα/TNFR1 dual catabolic and pyroptotic axes driving sarcopenia, highlighting environmental plasticizers as not solely a traditional geriatric but a broad public health risk.

An advantage of this investigation is its departure from only binary clinical diagnoses or functional proxies (e.g., grip strength (18), walking speed (19) used in previous environmental epidemiology. While studies like Yoon et al. (19) paradoxically associated higher phthalate concentrations with faster walking speeds due to reverse causality artifacts, our study evaluated the continuous Appendicular Skeletal Muscle Mass Index (ASMI) derived from DXA. Using advanced qgcomp and BKMR models, we captured early-stage subclinical atrophy, suggesting that mixed phthalate exposure significantly erodes continuous ASMI. Furthermore, contemporary data reflects critical shifts in industrial chemical usage. While earlier surveillance (NHANES 1999-2006) highlighted MBZP as a primary risk factor (16), our mixture models consistently identified MBP as the predominant toxicological driver in modern populations. By rigorously excluding major metabolic confounders, we demonstrated that MBP uniquely maintained its high pathogenic risk profile, whereas other low-molecular-weight phthalates lost statistical significance. Furthermore, our models revealed the impact of collinearity confounding among highly correlated metabolites. While single-pollutant models obscured the role of MECPP, the BKMR model retained it with a high conditional posterior inclusion probability (Cond PIP = 1.0) when controlling for co-exposures. Given its lack of a negative univariate trend, MECPP likely functions as a critical conditional covariate or confounding adjustment factor within the mixture framework, rather than a direct pathogenic driver.

This multi-model approach also corrected historical demographic artifacts. Wang et al.’s conclusion that no significant gender interaction was observed (17). Other logistic regression models suggested females were most vulnerable to phthalate-induced muscle loss (16). However, this could be largely a statistical illusion driven by lower baseline muscle mass of females making the diagnostic threshold easier to cross. This study revealed through qgcomp model that males are the primary victims of environmental toxin exposure, thereby revising traditional perceptions of gender susceptibility. Importantly, our subgroup analyses overturned the “only youth sensitivity” hypothesis (17, 42), demonstrating profound vulnerability in the >40 age demographic (**Figure 1A**), thereby supporting a cumulative exposure and age-related resilience decline model. Moreover, restricted cubic spline (RCS) analysis revealed a linear dose-response relationship without a discernible safety threshold (**Supplementary Figure 2**), suggesting that even low-level exposure poses a continuous threat to muscle homeostasis. Crucially, unlike other observational studies whose explanations of mechanisms remain speculative, we conducted further mediation analyses. we identified the inflammation and oxidative stress marker Uric Acid to High-Density Lipoprotein Ratio (UHR) (26) as pivotal mediator.

A further contribution of the present study is the identification of the potential molecular initiating event dictating DBP toxicity in skeletal muscle. Historically, the toxicological framework for phthalate esters has categorized them almost exclusively as classic endocrine disruptors that exert their effects internally through nuclear receptors (e.g., PPARs) (43). While nuclear receptor binding accounts for widespread, slow-acting metabolic disruption, it cannot fully explain the acute, localized, and highly inflammatory tissue destruction observed in DBP-exposed skeletal muscle. Through an integration of network pharmacology, transcriptome and molecular docking simulations, we hypothesize DBP and its primary metabolite MBP may operate as structural pseudo-ligands, predictively capable of direct physical interactions with the primary trigger TNFα active pocket with high affinity, potentially stabilizing the active homotrimer of TNF-α, or altering the conformation to increase binding affinity to TNFR1 but not TNFR2, effectively “hijacking” the TNFR1 signaling axis. Because TNFR1 uniquely contains an intracellular Death Domain (DD) that drives inflammation and cell death, whereas TNFR2 lacks a DD. this specific receptor selectivity may partly explains the subsequent catabolic cascade, including UPS overactivation, autophagy, and pyroptosis. Although our epidemiological models identified the metabolite MBP as the primary risk factor, we utilized the parent compound DBP in our experimental models. Pharmacokinetically, orally administered DBP is rapidly hydrolyzed by gastrointestinal and serum esterases into MBP *in vivo*, meaning skeletal muscle is predominantly exposed to this active metabolite. While such robust systemic metabolism is limited in *in vitro* monocultures, our molecular docking simulations suggest that both DBP and MBP predictively share the same TNFα-binding capacity, effectively driving the same receptor-mediated toxicological network. This interaction triggers the dual activation of both canonical and non-canonical NF-κB pathways (**Figure 5**). Unlike previous observations restricted to macrophage cytokine release (44), we provide the evidence of DBP initiating muscle breakdown via this receptor-mediated axis.

Downstream of this receptor activation, the signaling cascade amplifies the ubiquitin-proteasome system (UPS) and autophagy, evidenced by the induction of E3 ligases MuRF1, MAFbx, p53 and Beclin1 (45, 46). Crucially, our data distinguishes this toxicity from simple protein degradation by identifying pyroptosis as a central pathogenic mechanism (47). This inflammatory necrosis creates a vicious cycle that exacerbates tissue injury. In parallel, Oil Red O staining revealed extensive intramyocellular lipid deposition. This lipotoxic phenotype is likely driven by a dual mechanism: a severe decline in mitochondrial fatty acid oxidation resulting from the observed shift from oxidative type IIA to glycolytic type IIB fibers, and concurrent cellular reprogramming. This lipotoxic environment not only impairs insulin signaling but also compromises regenerative capacity (48), as evidenced by the depletion of Pax7^+^ satellite cells and the induction of adipogenic genes. This suggests that muscle stem cells are diverted from myogenesis toward adipogenic trans-differentiation or senescence.

Given that oxidative stress orchestrates the TNF/NF-κB/NLRP3 cascade (49), reversing this pathology requires an intervention capable of simultaneously neutralizing oxidative stress and silencing the inflammatory feedback loop. To address this, the study evaluates Morroniside, an iridoid glycoside with known antioxidant and anti-inflammatory properties (24). Morroniside demonstrated vastly superior, comprehensive protective efficacy. Intervention with Morroniside significantly attenuated these pathological changes, not only by restores the cellular antioxidant defense architecture but by attenuating the transcription of inflammatory cytokines. Thus, Morroniside acts as an upstream inhibitor, preventing the phenotypic shift toward adipogenesis and proteolysis, and critically, blocks pyroptosis, thereby effectively alleviating DBP-induced atrophy.

While our multi-dimensional approach provides a robust chain of evidence, this study represents an initial step in characterizing environmental sarcopenia. Additionally, the experimental dosages (50 and 250 mg/kg) utilized in our murine models are higher than average environmental trace levels, as they are designed to simulate lifelong cumulative burdens within a compressed experimental timeframe. While this dosage gap is a recognized limitation, these concentrations remain toxicologically relevant when contextualizing extreme real-world scenarios, such as intensive occupational exposures or localized dermal absorption from high-concentration cosmetics (up to 24,304 µg/g) (10). The cross-sectional nature of the NHANES analysis limits definitive causal inferences, necessitating validation through long-term longitudinal cohorts to confirm the “aging-accelerator” potential of DBP. Furthermore, although molecular docking and biological assays strongly support the “Molecular Mimicry” hypothesis, direct structural visualization of the DBP-TNFα-TNFR1 ternary complex remains to be achieved. Future investigations employing biophysical binding assays (such as Surface Plasmon Resonance or Isothermal Titration Calorimetry) and high-resolution techniques, such as cryo-electron microscopy, will be essential to map the precise atomic interactions defining this mechanism.

In conclusion, the present investigation broadens the clinical understanding of sarcopenia: rather than solely a passive hallmark of aging, it also manifests as a targeted, environment-driven metabolic pathology threatening a wider population, including the young. Ultimately, these findings serve as an urgent toxicological warning: securing the skeletal muscle health of young and aging populations requires not only advanced pharmacological interventions but a massive paradigm shift in environmental policy to eliminate the silent, structural degradation caused by modern environment exposures.

## Data Availability

The data presented in the study are deposited in the NCBI Sequence Read Archive (SRA) repository, accession number PRJNA1455402 (https://www.ncbi.nlm.nih.gov/bioproject/PRJNA1455402). The NHANES data presented in the study are deposited in the National Center for Health Statistics repository (https://www.cdc.gov/nchs/nhanes/). The other original contributions presented in the study are included in the article/**Supplementary Material**. Further inquiries can be directed to the corresponding authors.

## Supplementary material

Supplementary Figure 1 Flowchart of participant selection from NHANES 2011–2018 database. The sequential exclusion process resulted in a final analytical sample of 3,514 participants. Reasons for exclusion included: age < 20 years, missing urinary phthalate metabolites data, missing appendicular lean mass (ALM) or height data, and extreme urinary creatinine concentrations (< 10 or > 500 mg/dL). Abbreviations: NHANES, National Health and Nutrition Examination Survey; BMI, body mass index; PIR, poverty income ratio.

Supplementary Figure 2 Dose-response relationships between urinary phthalate metabolites and sarcopenia risk evaluated by restricted cubic spline (RCS) regression. The non-linear associations are presented for (A) MBP, (B) MCNP, (C) MECPP, and (D) MEHP. Solid lines represent the estimated odds ratios (ORs), and shaded areas indicate the corresponding 95% confidence intervals (CIs). The reference value (OR = 1.00) is indicated by the dashed horizontal line. For each metabolite, four levels of adjustment were applied: Model I: Factored in basic sociodemographic parameters, including age, gender, racial/ethnic background, educational attainment, marital standing, and PIR. Model II: Incorporated all Model I variables alongside lifestyle and clinical factors (BMI, smoking habits, alcohol use, stroke, diabetes, and hypertension). Model III: The fully adjusted framework, adding total energy and protein consumption, physical activity, chronic kidney disease (CKD), and congestive heart failure to Model II. *P*-overall and *P*-non-linear values are provided for each model to indicate the statistical significance and nature of the dose-response relationship.

Supplementary Figure 3 Spearman correlation heatmap of 13 urinary phthalate metabolites. Individual values within the matrix represent the Spearman correlation coefficients (r). The visual dimensions-specifically circle diameter and color saturation-illustrate the magnitude of these associations. Positive relationships are rendered in blue, whereas inverse associations are depicted in red. Finally, a central diagonal axis of perfect positive correlations (r = 1.00) confirms the baseline self-identity of each plotted biomarker.

Supplementary Figure 4 Population-specific risks and component interactions within the phthalate mixture. **(A)** Subgroup Cumulative Effects: Estimated risk of sarcopenia associated with the phthalate mixture across quantiles for the total population (Sarcopenia) and specific subgroups (Male, Female, Age >40, and Age 20-40). The downward trends with 95% posterior credible intervals indicate a cumulative reduction in muscle mass (ASMI) as mixture concentrations increase, with higher vulnerability observed in the Age >40 group. **(B)** Bivariate Exposure-Response Matrix: This matrix explores the potential interactive effects between individual phthalate metabolites on ASMI. Each plot displays the estimated effect of one metabolite (horizontal axis) when a second metabolite (indicated by the row/column labels) is fixed at its 10th (blue), 50th (orange), or 90th (red) percentile, while all other mixture components are held at their median levels. The overlapping nature of the blue, orange, and red curves indicates that the relationship between individual phthalates and ASMI is relatively independent of the concentrations of other mixture components, suggesting a lack of strong linear interactions.
