# Supplementary figures and images for: Dibutyl phthalate induces sarcopenia via TNFα/TNFR1-mediated proteolytic and pyroptotic axes: evidence from NHANES and experimental models
Source: Front Immunol. 2026 Jun 12;17:1853039. doi: 10.3389/fimmu.2026.1853039 (PMC13303147; doi:10.3389/fimmu.2026.1853039)

Fig. S1

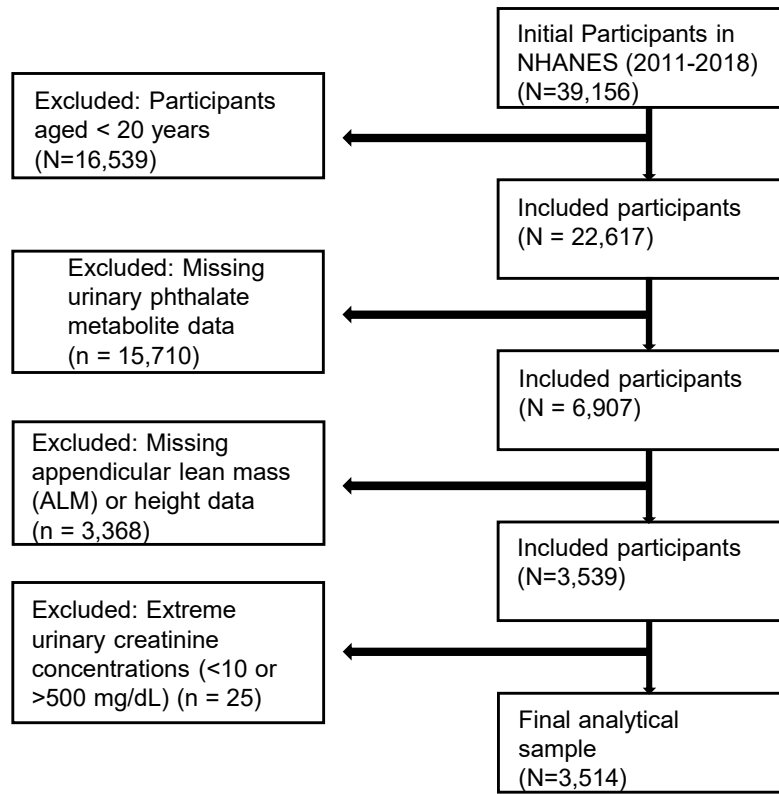

Fig. S2

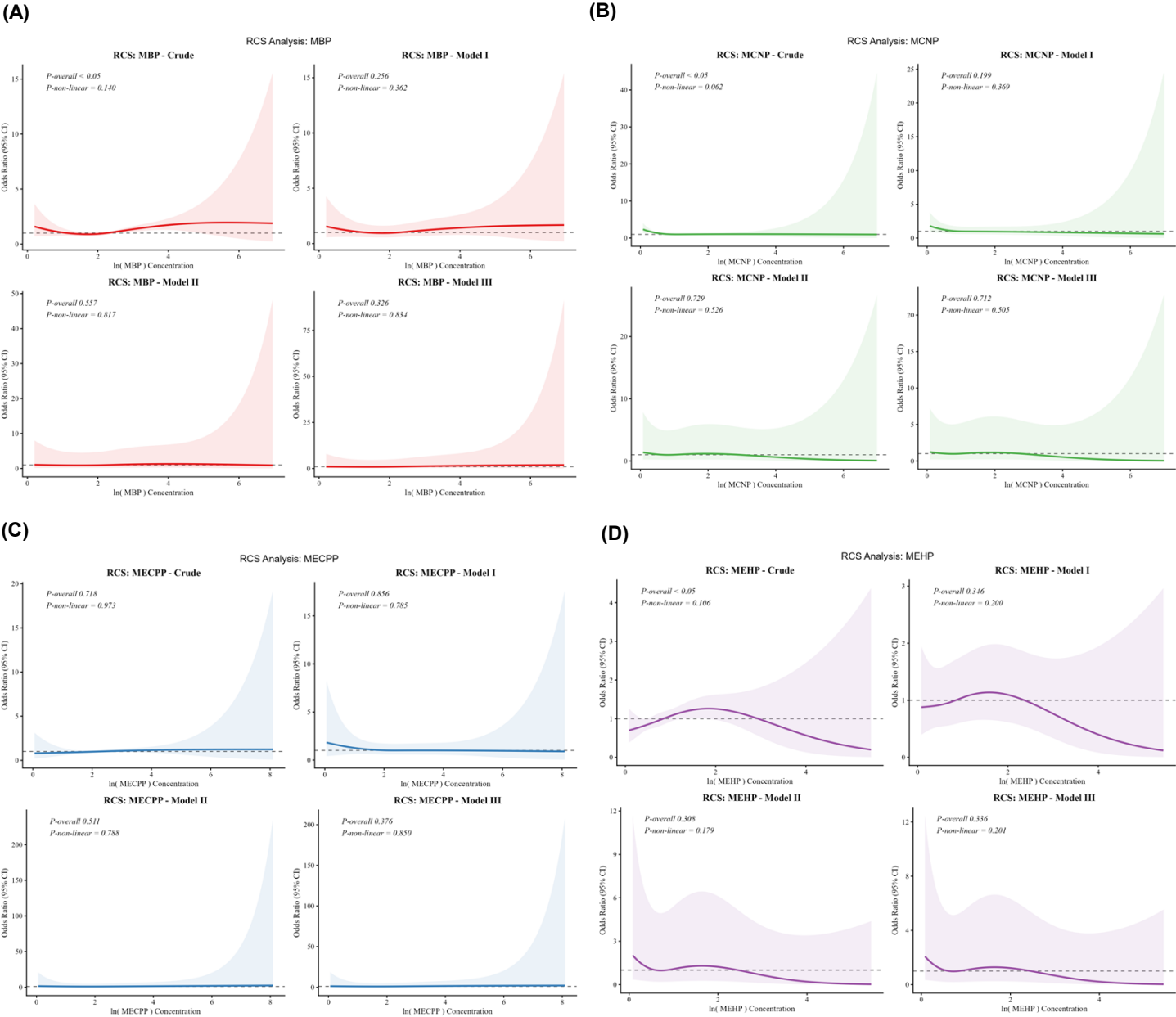

Fig. S3

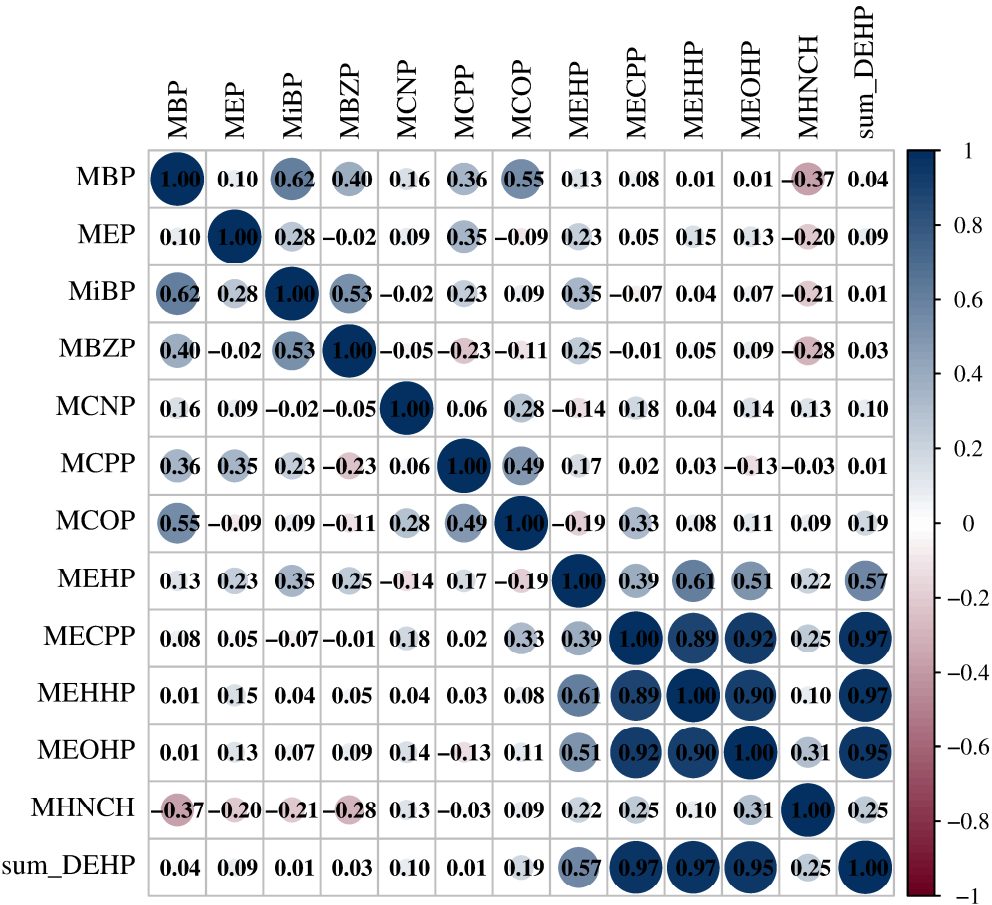

Fig. S4

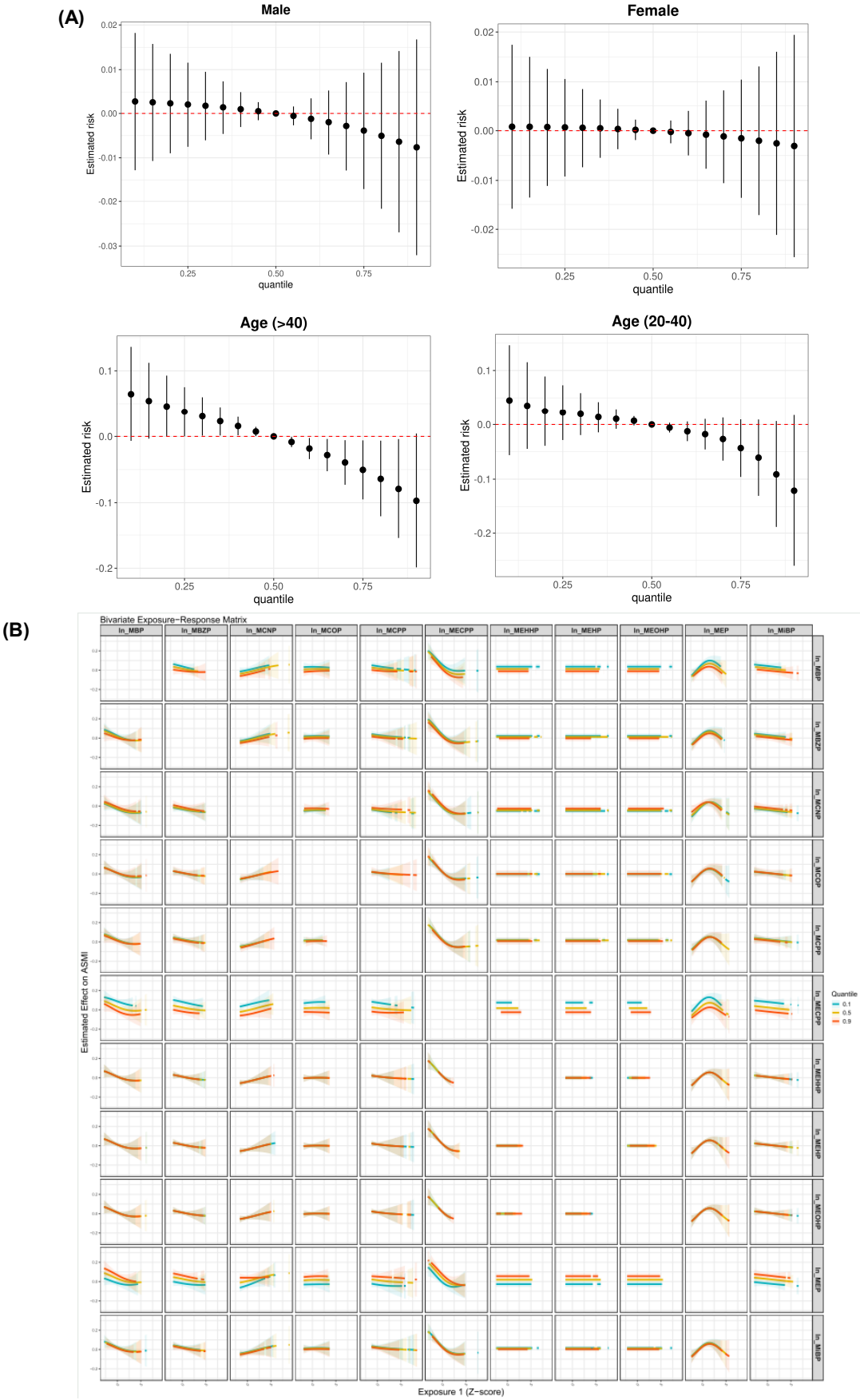

Supplement: Supplementary Figure 1 — Flowchart of participant selection from NHANES 2011–2018 database. The sequential exclusion process resulted in a final analytical sample of 3,514 participants. Reasons for exclusion included: age < 20 years, missing urinary phthalate metabolites data, missing appendicular lean mass (ALM) or height data, and extreme urinary creatinine concentrations (< 10 or > 500 mg/dL). Abbreviations: NHANES, National Health and Nutrition Examination Survey; BMI, body mass index; PIR, poverty income ratio. [file SupplementaryFile1.pdf]
