# Supplementary material for: Dibutyl phthalate induces sarcopenia via TNFα/TNFR1-mediated proteolytic and pyroptotic axes: evidence from NHANES and experimental models
Source: Front Immunol. 2026 Jun 12;17:1853039. doi: 10.3389/fimmu.2026.1853039 (PMC13303147; doi:10.3389/fimmu.2026.1853039)
Supplement: Supplementary file 2 [file SupplementaryFile2.docx]

# 1. Supplement Materials and Methods

## 1.1. Study Population

Our analytical cohort was derived from the National Health and Nutrition Examination Survey (NHANES), spanning four consecutive biennial cycles between 2011 and 2018. Administered by the CDC, this cross-sectional program provides a representative sample of the US population. We limited our analysis to individuals at least 20 years of age. A stepwise exclusion process was applied, removing subjects who lacked: (1) complete urinary phthalate metabolite measurements; or (2) the necessary DXA-derived appendicular lean mass (ALM) and height records required for sarcopenia classification. Furthermore, to guarantee the reliability of spot urine analyses, individuals presenting with abnormal urinary creatinine levels (under 10 mg/dL or over 500 mg/dL) were omitted. Ultimately, 3,514 adults were retained for final evaluation. All survey procedures received ethical clearance from the NCHS Research Ethics Review Board (under protocols #2011-17 and #2018-01), with written informed consent provided by every participant.

## 1.2. Exposure Assessment and Covariates

To address the right-skewed nature of the biomarker data, we applied a natural logarithmic (ln) transformation to the concentrations of the 13 evaluated urinary phthalate metabolites **(Table S2)**. We then divided these continuous variables into four equal groups to evaluate dose-response trends, establishing the bottom quartile as our baseline reference group. Guided by established research and directed acyclic graphs (DAGs), we incorporated a comprehensive set of confounding variables. Demographic indicators comprised age, gender, racial/ethnic background, educational attainment, marital standing, and the poverty income ratio (PIR). Health and behavioral metrics factored into the models were BMI, smoking habits, alcohol use, and a history of stroke, diabetes, or hypertension. For our most rigorously adjusted models, we further controlled for total caloric and protein consumption, physical activity levels, chronic kidney disease (CKD), and congestive heart failure (CHF). Finally, we utilized multiple imputation by chained equations (MICE) to resolve any missing covariate data, thereby preserving our analytical sample size and reducing potential selection bias.

## 1.3. Outcome definition

To classify participants' sarcopenia status, we relied on the Appendicular Skeletal Muscle Mass Index (ASMI). This metric is derived by taking the total appendicular skeletal muscle mass (ASM) and normalizing it against the participant's height squared (m²). We adopted the EWGSOP2 (European Working Group on Sarcopenia in Older People) thresholds to identify individuals with low muscle mass, applying cutoffs of ASMI below 7.0 kg/m² for males and under 5.5 kg/m² for females(Cruz-Jentoft et al., 2019). Furthermore, to capture granular fluctuations in muscle mass, our linear regression models

incorporated ASMI as a continuous dependent variable.

## 1.4. Epidemiological statistical analysis

All statistical computations were executed within the R environment (version 4.5.1). We applied sample weights to ensure the data remained nationally representative. Weighted logistic regression models estimated odds ratios (ORs) for sarcopenia, while linear regression assessed associations with continuous ASMI. Restricted cubic splines (RCS) characterized potential non-linear dose-response relationships. To tackle the challenges of concurrent exposure and minimize multicollinearity, we implemented Weighted Quantile Sum (WQS) regression. Alongside this, quantile g-computation (qgcomp) was utilized to ascertain the collective impact of the biomarker mixture. We also deployed Bayesian Kernel Machine Regression (BKMR) to elucidate complex interactions and non-linear dependencies, treating any variable with a Posterior Inclusion Probability (PIP) greater than 0.5 as a key contributor. Finally, mediation analysis examined inflammation and oxidative stress markers (Oxidative Balance Score (OBS) (Zhou et al., 2025), Uric acid to High-density lipoprotein cholesterol Ratio (UHR) (Mao et al., 2025) as potential pathways. We estimated indirect effects using 1,000 Monte Carlo simulations.

Reference

Cruz-Jentoft, A.J., Bahat, G., Bauer, J., Boirie, Y., Bruyère, O., Cederholm, T., Cooper, C., Landi, F., Rolland, Y., Sayer, A.A., Schneider, S.M., Sieber, C.C., Topinkova, E., Vandewoude, M., Visser, M., Zamboni, M., Writing Group for the European Working Group on Sarcopenia in Older People 2 (EWGSOP2), and the Extended Group for EWGSOP2, 2019. Sarcopenia: Revised european consensus on definition and diagnosis. Age Ageing 48, 16–31. https://doi.org/10.1093/ageing/afy169

Mao, H., Zhang, X., Huang, S., Lin, T., Chen, Z., 2025. Relationship between uric acid to high-density lipoprotein cholesterol ratio and sarcopenia in NHANES: Exploring the mediating role of bilirubin and association with all-cause mortality. Front Nutr 12, 1560617. https://doi.org/10.3389/fnut.2025.1560617

Zhou, Q., Zhou, S., Chen, Z.-X., Zhou, W.-J., Su, J., Wang, Y., 2025. Associations of dietary oxidative balance score with sarcopenia in adults: An NHANES-based cross-sectional study. Nutr Metab (Lond) 22, 6. https://doi.org/10.1186/s12986-025-00894-4
